# Chemotherapy-related cachexia is associated with mitochondrial depletion and the activation of ERK1/2 and p38 MAPKs

**DOI:** 10.18632/oncotarget.9779

**Published:** 2016-06-02

**Authors:** Rafael Barreto, David L. Waning, Hongyu Gao, Yunlong Liu, Teresa A. Zimmers, Andrea Bonetto

**Affiliations:** ^1^ Department of Surgery, Indiana University School of Medicine, Indianapolis, IN 46202, USA; ^2^ Simon Cancer Center, Indiana University School of Medicine, Indianapolis, IN 46202, USA; ^3^ Department of Medicine, Indiana University School of Medicine, Indianapolis, IN 46202, USA; ^4^ Department of Medical and Molecular Genetics, Center for Computational Biology and Bioinformatics, Indiana University School of Medicine, Indianapolis, IN 46202, USA; ^5^ IUPUI Center for Cachexia Research, Innovation and Therapy, Indianapolis, IN 46202, USA

**Keywords:** chemotherapy, cachexia, muscle wasting, MAPKs, mitochondria

## Abstract

Cachexia affects the majority of cancer patients, with currently no effective treatments. Cachexia is defined by increased fatigue and loss of muscle function resulting from muscle and fat depletion. Previous studies suggest that chemotherapy may contribute to cachexia, although the causes responsible for this association are not clear. The purpose of this study was to investigate the mechanism(s) associated with chemotherapy-related effects on body composition and muscle function. Normal mice were administered chemotherapy regimens used for the treatment of colorectal cancer, such as Folfox (5-FU, leucovorin, oxaliplatin) or Folfiri (5-FU, leucovorin, irinotecan) for 5 weeks. The animals that received chemotherapy exhibited concurrent loss of muscle mass and muscle weakness. Consistently with previous findings, muscle wasting was associated with up-regulation of ERK1/2 and p38 MAPKs. No changes in ubiquitin-dependent proteolysis or in the expression of TGFβ-family members were detected. Further, marked decreases in mitochondrial content, associated with abnormalities at the sarcomeric level and with increase in the number of glycolytic fibers were observed in the muscle of mice receiving chemotherapy. Finally, ACVR2B/Fc or PD98059 prevented Folfiri-associated ERK1/2 activation and myofiber atrophy in C2C12 cultures. Our findings demonstrate that chemotherapy promotes MAPK-dependent muscle atrophy as well as mitochondrial depletion and alterations of the sarcomeric units. Therefore, these findings suggest that chemotherapy potentially plays a causative role in the occurrence of muscle loss and weakness. Moreover, the present observations provide a strong rationale for testing ACVR2B/Fc or MEK1 inhibitors in combination with anticancer drugs as novel strategies aimed at preventing chemotherapy-associated muscle atrophy.

## INTRODUCTION

Cachexia, a devastating condition occurring in 22% to 55% of advanced colorectal cancer cases, is associated with marked loss of body weight and depletion of fat and muscle storage [1, 2]. Cachexia is primarily characterized by skeletal muscle and adipose tissue wasting, but is also associated with increased fatigue, poor performance status, reduced quality of life and high mortality [3, 4]. Available evidence suggests that chemotherapy (i.e. the use of cytotoxic and antiproliferative drugs) may play a key role in the development and sustainment of cachexia. Chemotherapy is frequently accompanied by numerous side effects, including nausea, diarrhea, anorexia. Among these, increased muscle weakness represents one of the most debilitating [5], affecting patient's quality of life and resulting in increased morbidity and mortality.

Despite the fact that the molecular mechanisms responsible for the development of cachexia have been studied for several decades, little is known about the effects of various cancer treatments on cachexia-related symptoms, and a plethora of conflicting observations are present in the literature. It has been suggested that several antineoplastic drugs may cause direct host cell modification as well as induce a negative nitrogen balance in healthy animals and in tumor hosts [6]. For instance, Damrauer et al. reported that several chemotherapeutics, including cisplatin, CPT-11, adriamycin, and etoposide, may directly cause muscle wasting via activation of the NF-κB pathway, and suggested that these effects were independent of their inhibition of tumor growth as well as commonly implicated ubiquitin-proteasome system or indirect effects via production of pro-inflammatory cytokines, such as IL-1β, IL-6 and TNF [7]. The same chemotherapeutics were also shown to induce oxidative stress, thereby promoting tissue injury [7–9]. In contrast with these observations, previous studies found that cisplatin, part of the standard care of treatment of lung, ovary, testicular and bladder cancers, activates inflammation and ubiquitin-dependent catabolism and down-regulates muscle anabolism in mice, thus leading to weight loss associated with loss of adipose tissue and muscle mass [10, 11]. Cisplatin was also show to up-regulate the expression of myostatin, a member of the TGF-beta family of growth factors also known to negatively regulate muscle mass in the occurrence of cancer, thus contributing to muscle wasting via activation of proteasome-dependent muscle catabolism [11–13]. Similarly, sorafenib, a multi-kinase inhibitor successfully tested for the treatment of several kinds of pre-clinical tumor models [14], was shown to promote muscle wasting in association with the activation of the ubiquitin-and Ca^2+^-dependent proteolytic systems [15].

Regardless of the mechanisms of action, preserving muscle mass may also represent an important therapeutic strategy for the treatment of cachexia associated with chemotherapy administration. Indeed, several indications recommended the use of strategies aimed at antagonizing myostatin in order to enhance muscle anabolism and prevent muscle catabolism in cancer cachexia and to prolong life, even in the absence of direct effects on tumor growth [13, 16–19]. Analogously, administration of ghrelin, an endogenous ligand for the growth hormone secretagogue receptor (GHSR)-1a, was shown to protect against cisplatin-induced cachexia by promoting muscle anabolism in experimental animals [11]. To further support the idea that promoting muscle mass may benefit patients' tolerance to chemotherapy, it was also reported that cancer patients affected with muscle depletion (regardless of body weight) are more susceptible to developing severe drug-associated toxicity and show a poorer prognosis. In contrary, subjects with higher muscle mass or those who do not show sarcopenia at diagnosis are generally more resistant and may tolerate higher doses of chemotherapy that, in turn, would likely increase the chance of cure and disease-free survival [1, 20–23].

The purpose of this study was to investigate whether chemotherapeutics, such the ones that are utilized for the treatment of colorectal tumors, promote the development of cachexia. In order to do so, we tested *in vivo* chemotherapy regimens frequently used as preferred therapy for the treatment of colorectal cancers, in particular in the late metastatic stages of the disease, such as combinations of 5-fluorouracil (5-FU), leucovorin and either CPT-11 (i.e. Folfiri) or oxaliplatin (i.e. Folfox). We assessed muscle morphology and fiber size in the presence of anticancer compounds, effects on body composition and muscle strength, along with the modulation of chemotherapy-specific genes and proteins. We then investigated whether promoting muscle growth by using soluble Activin Receptor 2B (ACVR2B/Fc), a widely studied myostatin inhibitor, or whether preventing the activation of the MAPKs by administering PD98059, a MEK1 pharmacologic antagonist, in combination with Folfiri prevented the associated myofiber atrophy in C2C12 myotube cultures. The findings from this study suggest a potentially causative role for chemotherapy in the occurrence of muscle loss and weakness, and support the investigation of strategies making use of ACVR2B/Fc or MEK1 inhibitors in combination with anticancer drugs in order to prevent chemotherapy-associated cachexia.

## RESULTS

### *In vivo* chemotherapy administration causes adipose tissue and skeletal muscle weight loss as well as a transient decrease in food intake

**Figure 1:**
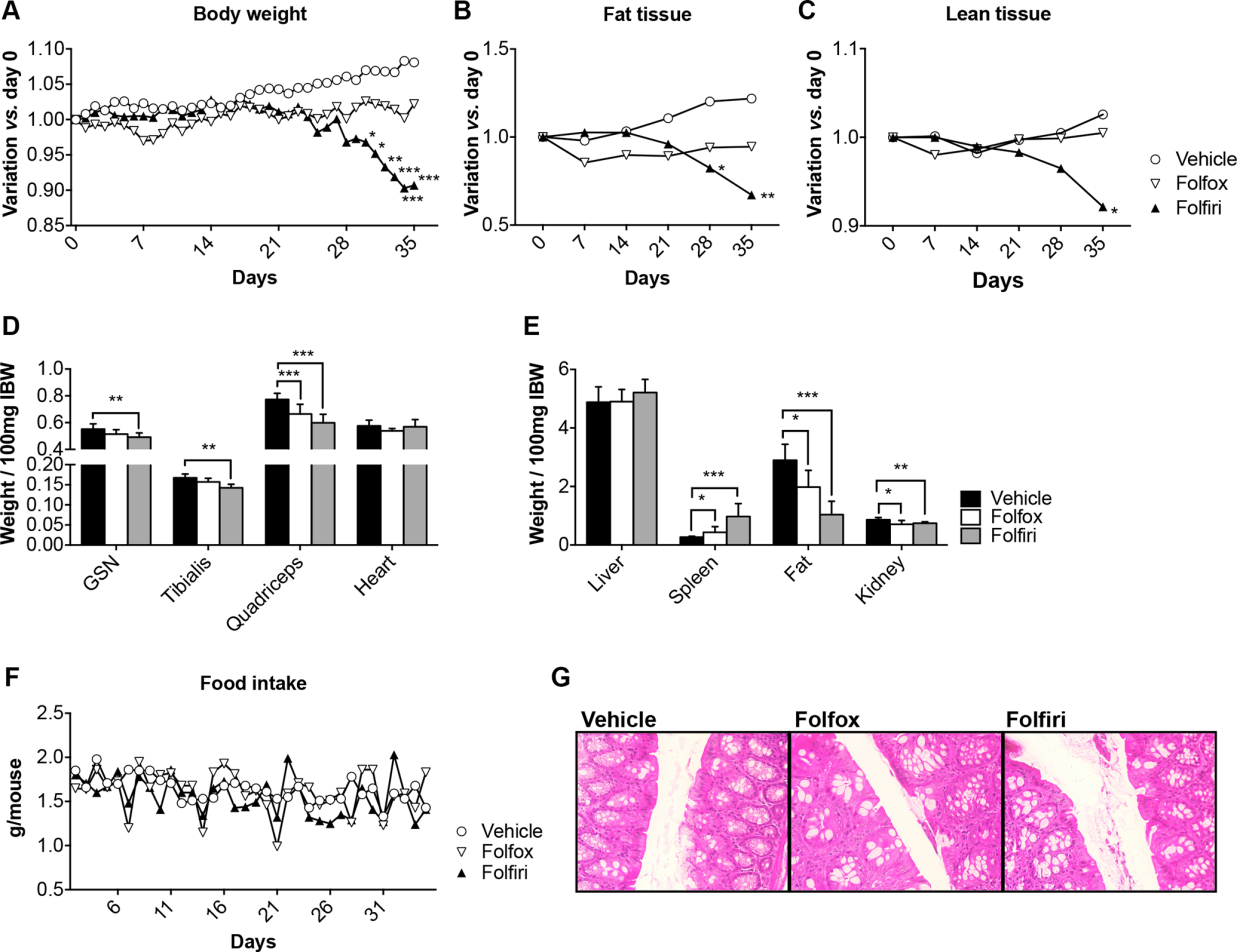
*In vivo* chemotherapy administration causes adipose tissue and skeletal muscle weight loss Body weights (**A**), body composition assessment (fat and lean tissues) performed by means of EchoMRI (**B–C**), muscle (**D**) and organ (**E**) weights in mice treated with chemotherapy for up to 5 weeks (*n* = 4–6). Weights were normalized to the Initial Body Weight (IBW) and expressed as weight/100mg IBW. Overall food intake over the 5-week experimental period (**F**). Representative gut morphology in vehicle- and chemotherapy-treated animals (**G**). FBW: Final Body Weight; GSN: Gastrocnemius; Gem: Gemcitabine. Significance of the differences: *p < 0.05; **p < 0.01; ***p < 0.001 *vs.* Vehicle.

In order to investigate whether chemotherapy was able to directly affect muscle mass growth and homeostasis, we administered Folfox or Folfiri chemotherapy regimens to adult (8-week old) male CD2F1 healthy mice (*n* = 8) for up to 5 weeks. Control animals were administered the vehicle only. We observed no changes in body weight over the first 3 weeks of treatment; however, the Folfiri-treated animals showed progressive body weight loss starting at week 4, while the mice receiving Folfox substantially maintained their initial body weight (Figure 1A). At the end of the 5-week period, the Folfiri-treated animals showed significant loss of body weight (about 10% *vs.* vehicle, *p* < 0.01; Figure 1A), consistent with marked loss of fat (Figure 1B) and lean tissue (Figure 1C). Notably, the administration of chemotherapy did not affect the overall body growth, as also supported by the absence of differences in the tibia length among Folfiri- and vehicle-treated animals (Figure S1). All the chemotherapy-treated animals showed marked quadriceps muscle wasting (−23% *vs.* vehicle, *p* < 0.001), while only the mice that received Folfiri also exhibited loss of gastrocnemius and tibialis anterior mass (Figure 1D). Interestingly, the decrease in muscle mass following chemotherapy treatment was not associated with a reduction in the overall mobility, as shown by the mouse activity monitoring (Figure S2). Further, no effects on cardiac muscle were observed (Figure 1D). Following chemotherapy treatment, splenomegaly and severe depletion of gonadal adipose tissue and kidney mass were recorded (Figure 1E). In order to establish whether these effects were associated with changes in food intake, food consumption was monitored daily. Some sudden drops in food intake were detected in the mice soon after the administration of chemotherapy, although no significant difference in the average consumption was reported over the whole experimental period (Figure 1F; AUC Vehicle = 53.97, Folfox = 53.09, Folfiri = 51.26). Further, no significant alterations were observed in the morphology of the gastrointestinal tract from mice that were administered the chemotherapeutics (Figure 1G).

### Folfiri-derived muscle atrophy associates with significant muscle weakness

**Figure 2:**
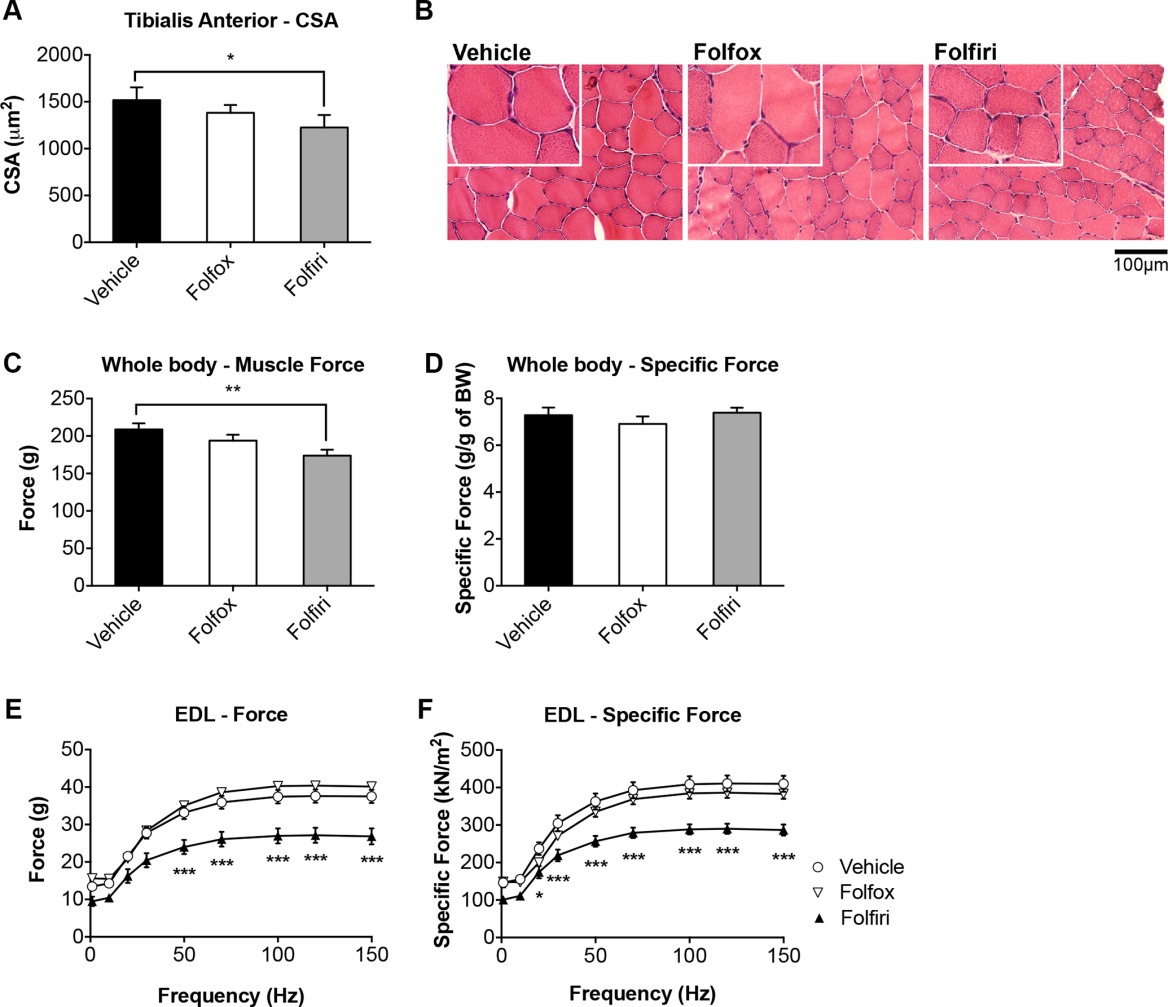
Folfiri-derived muscle atrophy associates with significant muscle weakness Muscle cross sectional area (CSA) in the tibialis muscle of mice exposed to chemotherapy (**A**) and tibialis anterior morphology (H&E staining) (**B**). Whole body grip strength, reported as peak force (**C**) and specific (normalized) force (**D**), was measured by taking advantage of a grip strength meter and expressed as the average of the three top pulls from each animal (*n* = 4–6). Ex-vivo muscle contractility was performed on EDL muscle excised from animals administered chemotherapy for up to 5 weeks (**E–F**). Data expressed as means ± SEM. Significance of the differences: *p < 0.05; **p < 0.01; ***p < 0.001 *vs.* Vehicle.

Unlike Folfox, Folfiri caused marked depletion of skeletal muscle mass, consistent with reduction of fiber size in the tibialis anterior muscle (−20% *vs.* vehicle, *p* < 0.05; Figure 2A–2B). The loss of muscle mass experienced following Folfiri administration was also associated with a reduction in muscle strength, assessed as whole body muscle force (−17% vs. vehicle, *p* < 0.01; Figure 2C). However, the evaluation of the specific whole muscle strength (normalized to the body weight) did not show any changes as compared to the control (Figure 2D). Notably, the *ex-vivo* muscle contractility assessment detected a marked decrease in EDL muscle force in the mice that were exposed to Folfiri for 5 weeks (Figure 2E–2F).

### The muscle of mice exposed to chemotherapy exhibits up-regulation of ERK1/2 and p38 MAPKs and down-regulation of mitochondrial proteins

**Figure 3:**
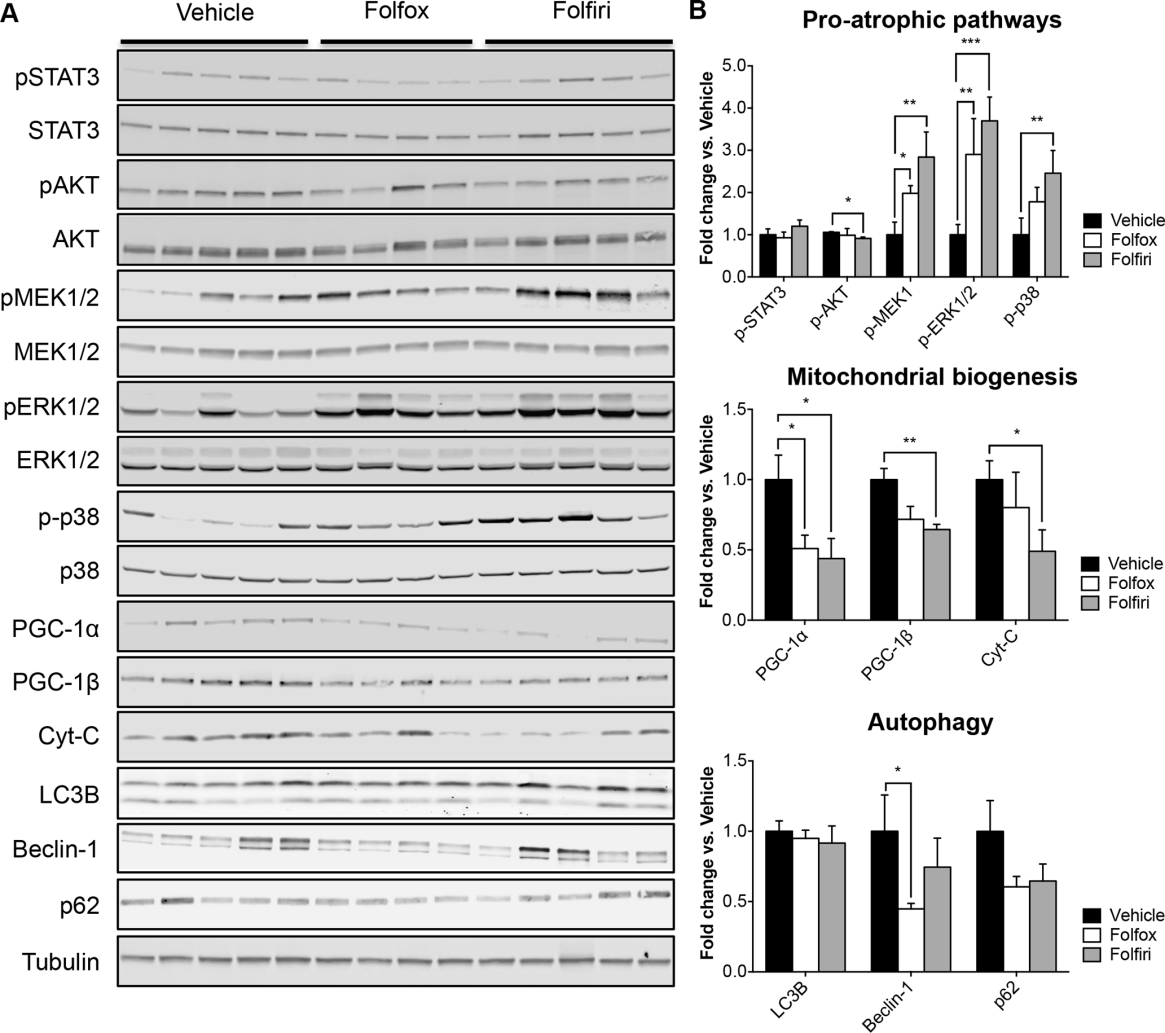
The muscle of mice exposed to chemotherapy exhibits up-regulation of ERK1/2 and p38 MAPKs and down-regulation of mitochondrial proteins Representative Western blotting (**A**) and quantification (**B**) for p-STAT3, STAT3, p-AKT, AKT, pMEK1/2, MEK1/2, p-ERK1/2, ERK1/2, p-p38, p38, PGC-1α, PGC-1β, Cytochrome C (Cyt-C), LC3B, Beclin-1 and p62 in muscle protein extracts from mice exposed to chemotherapy. Levels of phosphorylated proteins were normalized to the respective total protein expression. Tubulin was used as loading control. Data are expressed as Fold change *vs.* Vehicle and reported as means ± SEM. Significance of the differences: *p < 0.05; **p < 0.01; ***p < 0.001 *vs.* Vehicle.

In order to investigate whether the muscle phenotype observed following chemotherapy treatment was also associated with modulation of muscle-specific signaling pathways, we assessed the levels of a number of proteins known to be involved in the regulation of skeletal muscle growth. Interestingly, we observed modulation of several mediators of muscle atrophy or markers of mitochondrial biogenesis using Western blotting analysis performed on whole muscle protein extracts from mice exposed to chemotherapy, while contrary to previous reports in other experimental models [24, 25] muscle wasting did not seem to be associated with substantial modulation of autophagic degradation (Figure 3A–3B). In particular, all chemotherapy regimens showed hyperphosphorylation of both ERK1/2 and p38 MAPKs, thus suggesting that these signaling pathways might contribute to muscle wasting in the presence of anticancer treatments. These findings are consistent with previous data reported in other experimental conditions [26–28] (Figure 3A–3B). Of note, Folfiri administration was also associated with a significant reduction of muscle anabolism, as suggested by the decrease in AKT phosphorylation (Figure 3A–3B). Interestingly, mitochondrial proteins, such as PGC- 1α, PGC-1β and Cytochrome C, were also markedly decreased in the muscle of the chemotherapy-treated mice, thereby suggesting that mitochondrial alterations might also contribute to the development of muscle wasting and increased muscle weakness.

### Chemotherapy-derived muscle wasting is not associated with activation of ubiquitin-dependent proteolysis or on TGFβ expression

**Figure 4:**
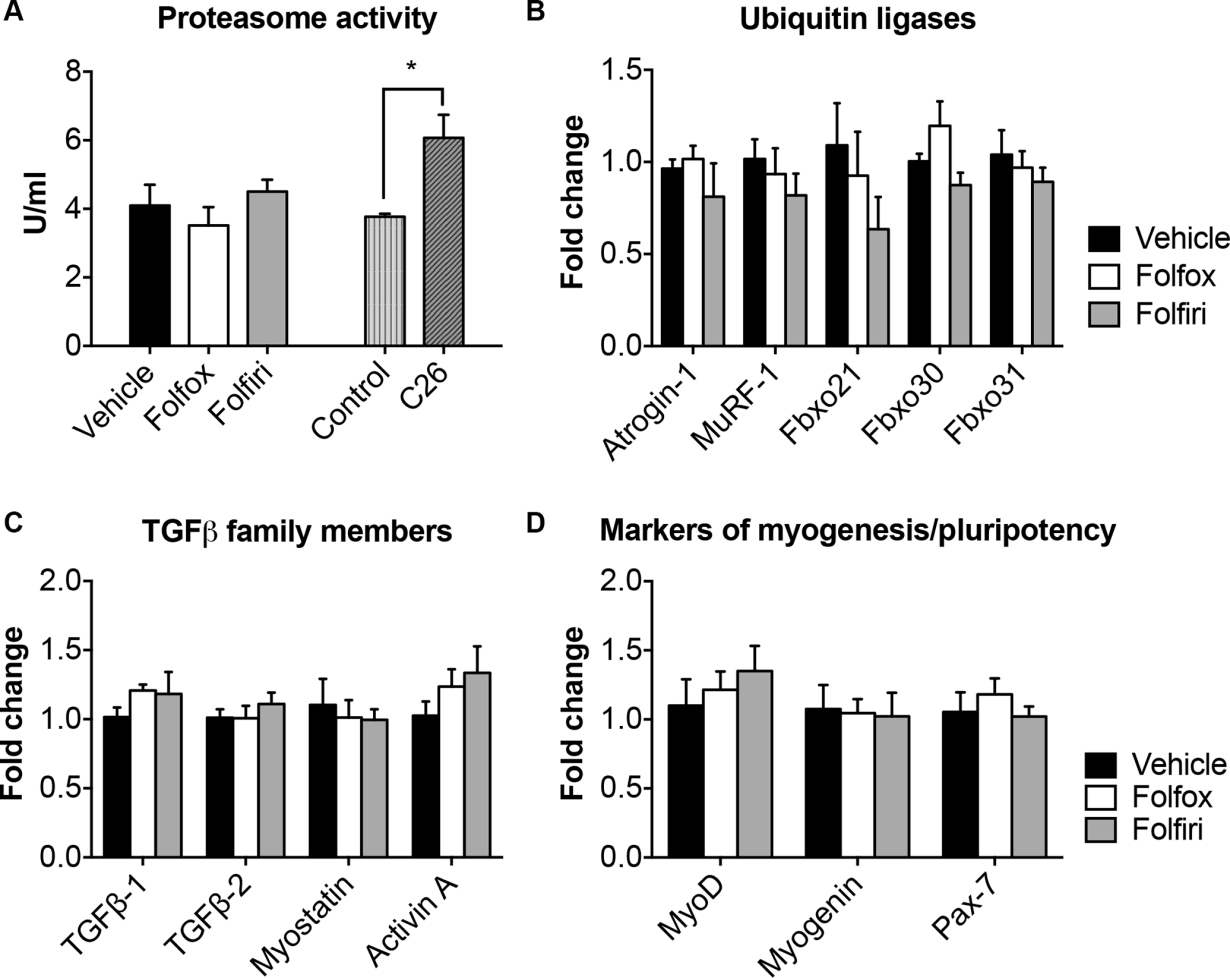
Folfiri-mediated muscle wasting is not associated with ubiquitin-dependent proteolysis or with increased expression of TGFβ-associated ligands or markers of myogenesis Chymotrypsin-like proteasome activity (U/ml) was performed on muscle from mice exposed to either Folfiri or Folfox and expressed as means ± SEM. Significance of the differences: *p < 0.05 *vs.* Vehicle (**A**). Gene expression levels for Atrogin-1, MuRF-1, Fbxo21 (SMART), Fbxo30 (MUSA1), Fbxo31 (**B**), TGF-β1, TGF-β2, myostatin, Activin A (**C**), MyoD, Myogenin and Pax-7 (**D**) was performed by qRT-PCR (Light Cycler 96, Roche, Indianapolis, IN). Gene expression was normalized to TBP levels. Data (fold change *vs.* vehicle) expressed as means ± SEM.

In order to test whether muscle wasting was associated with activation of muscle proteolysis, we measured the proteasome chymotrypsin activity in the quadriceps muscle of mice exposed to chemotherapy. No significant change was observed in the muscle of mice receiving either Folfox or Folfiri, unlike what shown in the quadriceps of mice bearing the C26 tumor (Figure 4A; see also [29, 30]). Further, gene expression analysis, performed by qRT-PCR, revealed no change in ubiquitin-ligases commonly overexpressed in conditions of muscle wasting, such as Atrogin-1, MuRF-1, Fbxo21 (SMART), Fbxo30 (MUSA1) and Fbxo31 [24], as measured in the skeletal muscle of mice exposed to chemotherapy for up to 5 weeks (Figure 4B). Similarly, TGFβ-associated ligands, such as TGFβ-1 and −2, myostatin and Activin A, previously shown to play a causative role in conditions of muscle wasting [12, 31, 32], were unchanged in the muscle of animals treated with either Folfox or Folfiri (Figure 4C). Interestingly, the expression of markers of myogenesis and pluripotency, such as MyoD, Myogenin and Pax-7, was unaffected by the treatment with chemotherapy (Figure 4D).

### Chemotherapy causes a shift towards more glycolitic fibers associated with marked depletion of mitochondria and aberrant muscle morphological features

**Figure 5:**
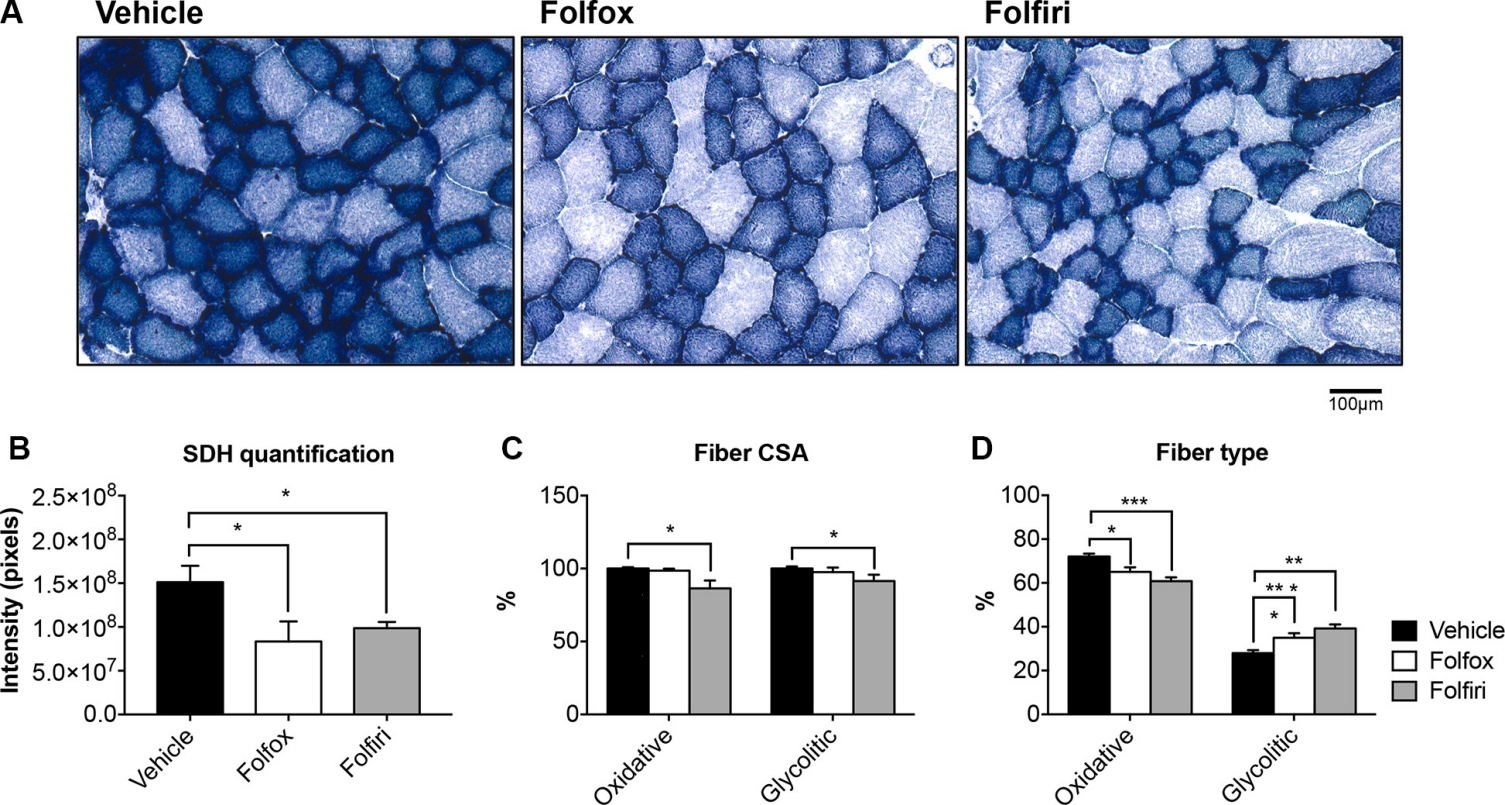
Chemotherapy causes marked reduction in mitochondrial activity and increase in the number of glycolytic muscle fibers Succinate dehydrogenase (SDH) staining was performed on 8 μm-thick sections from tibialis muscle frozen in liquid nitrogen-cooled isopentane (**A**). Quantification of signal intensity (expressed in pixels) (**B**), fiber-specific CSA (expressed as % of vehicle) (**C**) and number of oxidative (dark blue) and glycolytic (light blue) fibers (expressed as % of vehicle) (**D**) was assessed. Scale bar: 100 μm. Significance of the differences: *p < 0.05, **p < 0.01, ***p < 0.001 *vs.* Vehicle.

**Figure 6:**
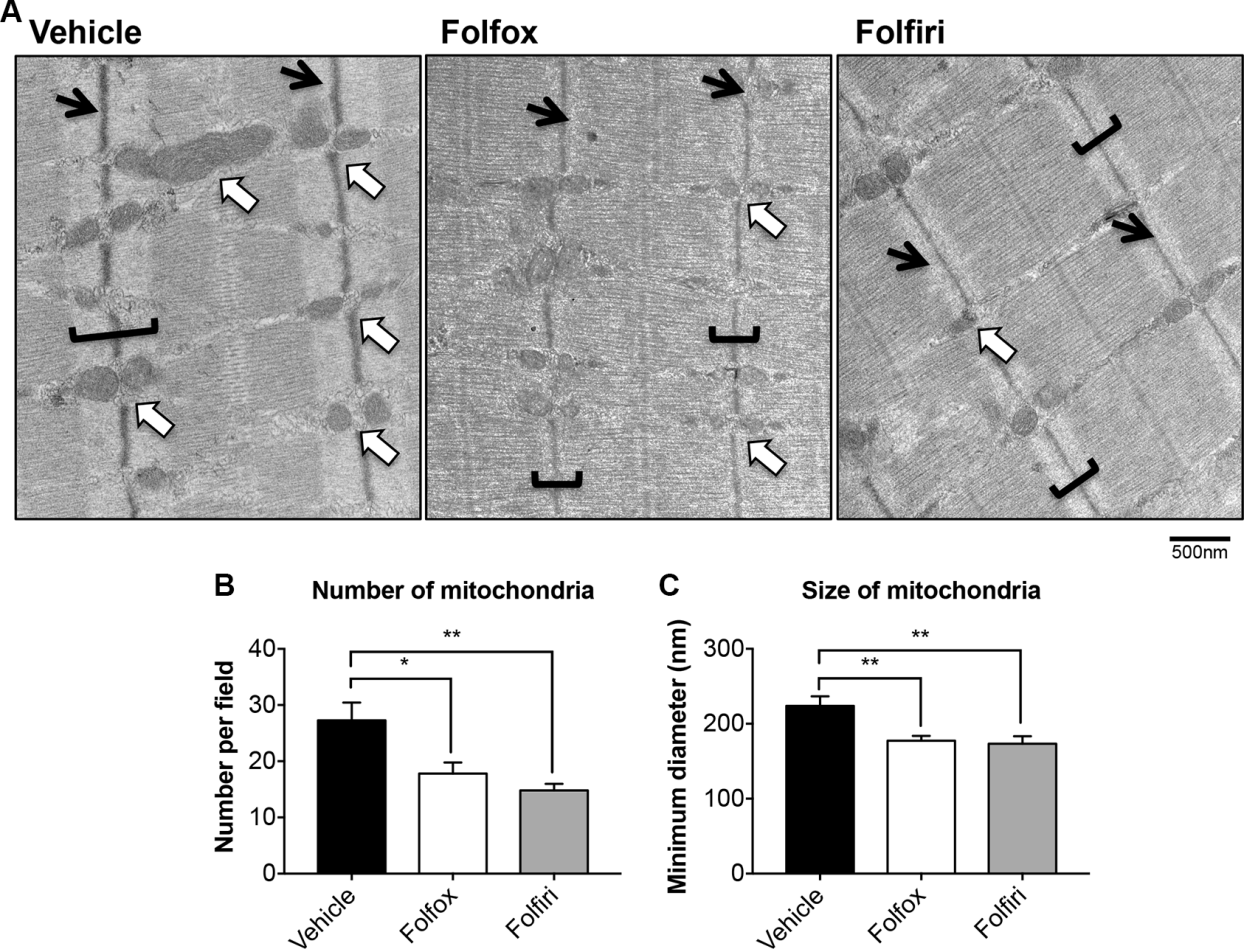
Chemotherapy causes depletion of mitochondria and aberrant muscle morphological features Electron microscopy micrographs (magnification: 30,000x) of EDL muscles from mice exposed to chemotherapy are reported in (**A**). White arrows indicate mitochondria. Black arrows indicate the Z-line. Brackets identify the I-bands. Scale bar: 500nm. Quantification of mitochondrial amount (number per field) (**B**) and size (minimum diameter, nm) (**C**) was performed. Significance of the differences: *p < 0.05, **p < 0.01 *vs.* Vehicle.

In line with the marked reduction in mitochondrial proteins observed in Figure 3, the succinate dehydrogenase (SDH) activity in the muscle of mice exposed to Folfox or Folfiri was markedly reduced compared to the vehicle-treated animals (Figure 5A–5B). This was also associated with a reduction in the size of both oxidative (dark blue) and glycolytic (light blue) fibers in the Folfiri treated animals (Figure 5C), in line with the CSA data displayed in Figure 2A–2B. Quite interestingly, chemotherapy was also shown to drive an oxidative to glycolytic fiber shift (Figure 5D), characterized by a reduction in oxidative fibers and a significant increase in the number of fibers associated with a more glycolytic metabolism in the tibialis muscle of mice exposed to either Folfox or Folfiri *vs.* Vehicle. Likewise, the TEM morphologic assessment of EDL muscles revealed major alterations at the sarcomeric level, with abnormalities consistent with fewer and smaller mitochondria (white arrows; Figure 6B–6C) and with markedly thinner Z-lines (black arrows) in the mice that received any of the chemotherapy regimens (Figure 6A). Similarly, the I-band, mainly constituted of thin actin filaments, appeared thinner or completely absent, particularly in the Folfox-treated animals (brackets; Figure 6A). Consistent with the mitochondrial toxicity observed following chemotherapy administration, increased levels of ROS were detected in the muscle of mice exposed to either Folfox or Folfiri (Figure S3).

### RNA-Seq analysis reveals down-regulation of mitochondrial metabolism and up-regulation of lipid transportation and energy metabolism

**Figure 7:**
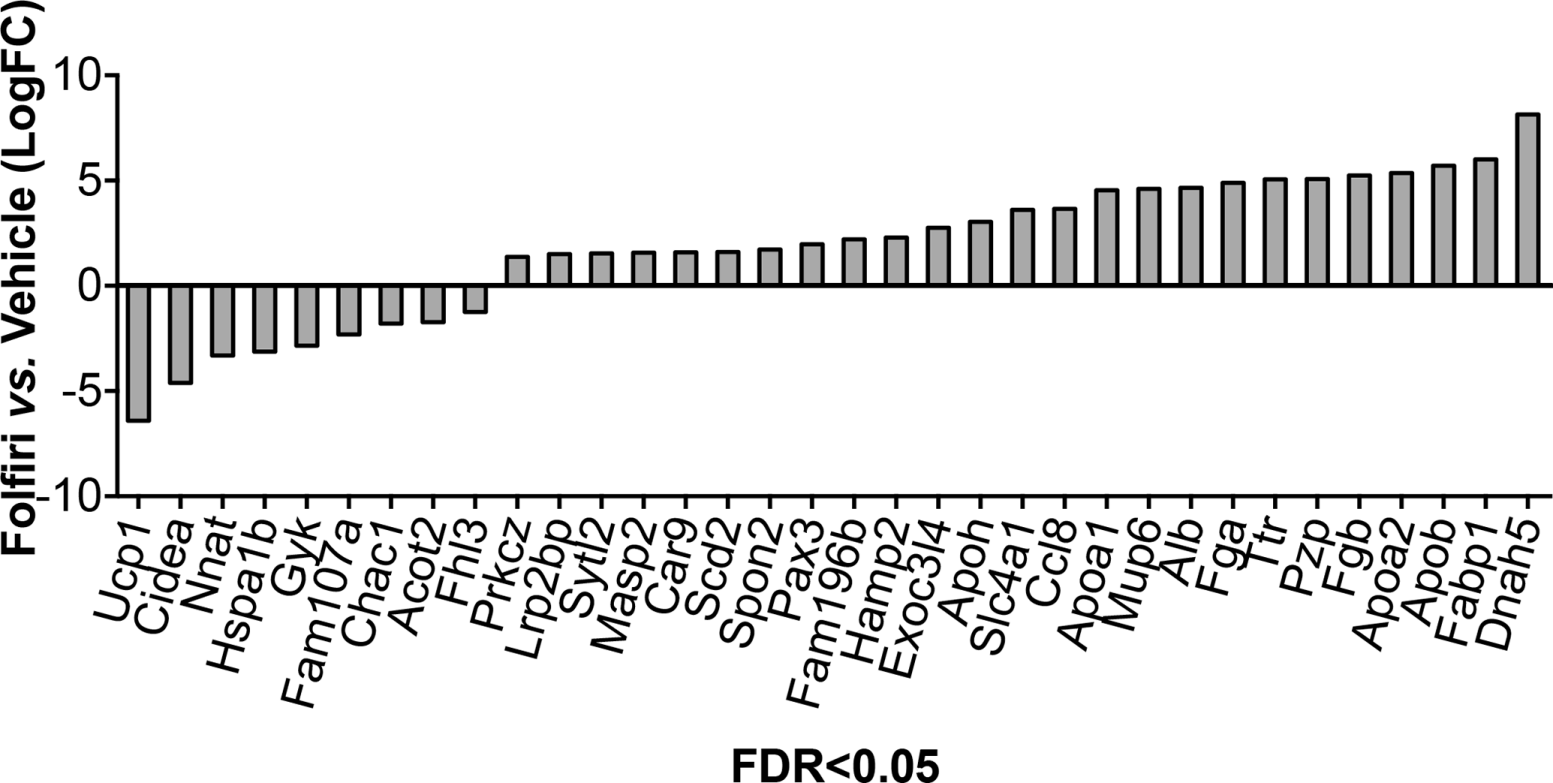
RNA-Seq analysis reveals down-regulation of mitochondrial metabolism and up-regulation of acute phase response proteins, lipid transportation and energy metabolism Next-Generation RNA-sequencing was performed on whole RNA extracted from skeletal muscle of Vehicle- and Folfiri-treated animals (*n* = 4). RNA-Seq reads were mapped to the mouse genome (mm 9). Only statistically significant differentially expressed genes (False Discovery Rate < 5%) between Vehicle- and Folfiri-exposed muscles are reported in the figure.

Gene expression profiling of quadriceps muscle, performed by means of RNA-Sequencing analysis, identified a limited set of genes that were significantly modulated (False Discovery Rate < 0.05) following Folfiri administration (Figure 7 and Table S1). Interestingly, the pathway analysis showed marked down-regulation of the regulators of mitochondrial metabolism Ucp1, Cidea1 and Acot2, as well as the marker of muscle cell proliferation/pluripotency Fhl3 (Figure 7 and Table S1). Further, we found increased expression of regulators of lipid metabolism and transport (such as Fabp1, Apoa1, Apob, Apoa2, Alb, Prkcz and Scd2) as well as acute phase response proteins (such as Alb, Fga and Fgb), and members of the PPAR signaling and markers of the Energy metabolism (such as Dnah5) were also detected (Figure 7 and Table S1).

### ACVR2B/Fc or PD98059 counteract Folfiri-mediated myofiber atrophy

**Figure 8:**
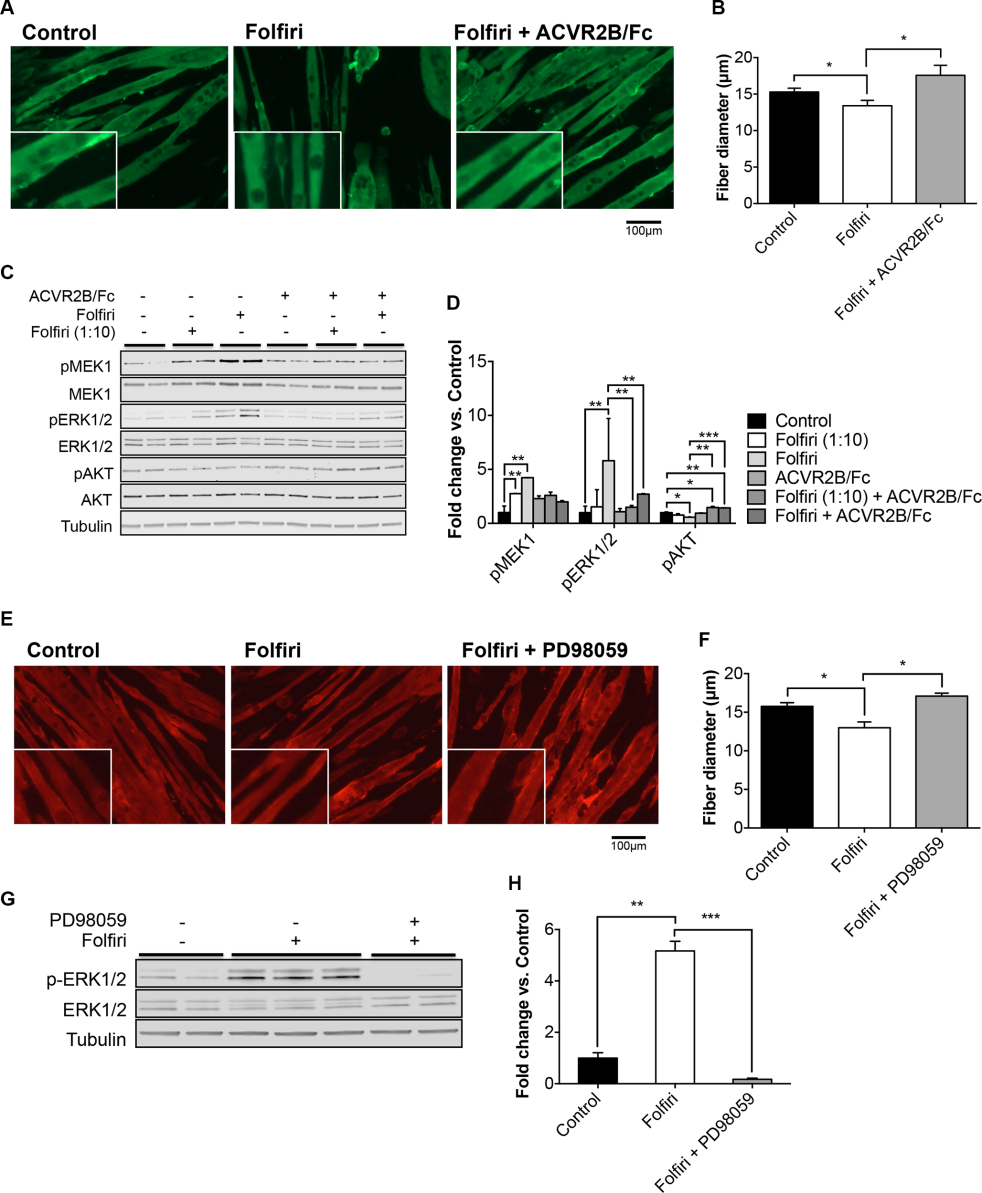
ACVR2B/Fc and PD98059 MEK1 inhibitor prevent Folfiri-associated muscle atrophy C2C12 murine myotubes were exposed to Folfiri in combination with either ACVR2B/Fc (10 μg/ml) or PD98059 (20 μM) for 48 h and later stained for Myosin Heavy Chain (MHC), visualized as green (**A**) or red (**E**) staining. Quantification of fiber size (*n* = 500–600) is reported in (**B–F**). Representative Western blotting (**C–G**) and quantification (**D–H**) for pMEK1/2, MEK1/2, pERK1/2, ERK1/2, pAKT and AKT in total protein extracts from C2C12 cultures. Tubulin was used as loading control. Scale bar: 100 μm. Data expressed as means ± SEM. Significance of the differences: *p < 0.05; **p < 0.01.

In order to evaluate whether strategies aimed at improving muscle mass or preventing Folfiri-associated activation of the MAPK signaling also result into a substantial protection of muscle mass, we exposed C2C12 murine myotubes to Folfiri, alone or in combination with either ACVR2B/Fc or PD98059, for 48h. Administration of ACVR2B/Fc (10 μg/ml) in the culture media was able to protect the myofibers from undergoing atrophy due to the Folfiri treatment (+31% *vs.* Folfiri, *p* < 0.05; Figure 8A–8B). In line with the observations reported in the *in vivo* model, Folfiri-dependent myotube atrophy (−13% *vs.* Control, *p* < 0.05) was associated with increased phosphorylation of the MAPK ERK1/2 and with reduced expression of the anabolic mediator AKT, already at a low dose (1:10) (Figure 8C–8D). Interestingly, the combined administration of ACVR2B/Fc not only restored the AKT-dependent anabolism, as suggested by the levels of phospho-AKT, but also prevented the activation of MEK1 and ERK1/2 (Figure 8C–8D). In order to investigate whether a more specific inhibition of ERK1/2 was able to prevent the Folfiri-dependent muscle protein loss, C2C12 myotubes were exposed to 20 μM MEK1 selective inhibitor PD98059 for up to 48h. Notably, the combined treatment showed a significant protection against Folfiri-mediated myofiber atrophy (+33% *vs.* Folfiri, *p* < 0.05; Figure 8E–8F), consistently with an almost complete inhibition of ERK1/2 activation, as suggested by the levels of phospho ERK1/2 (Figure 8G–8H).

## DISCUSSION

**Figure 9:**
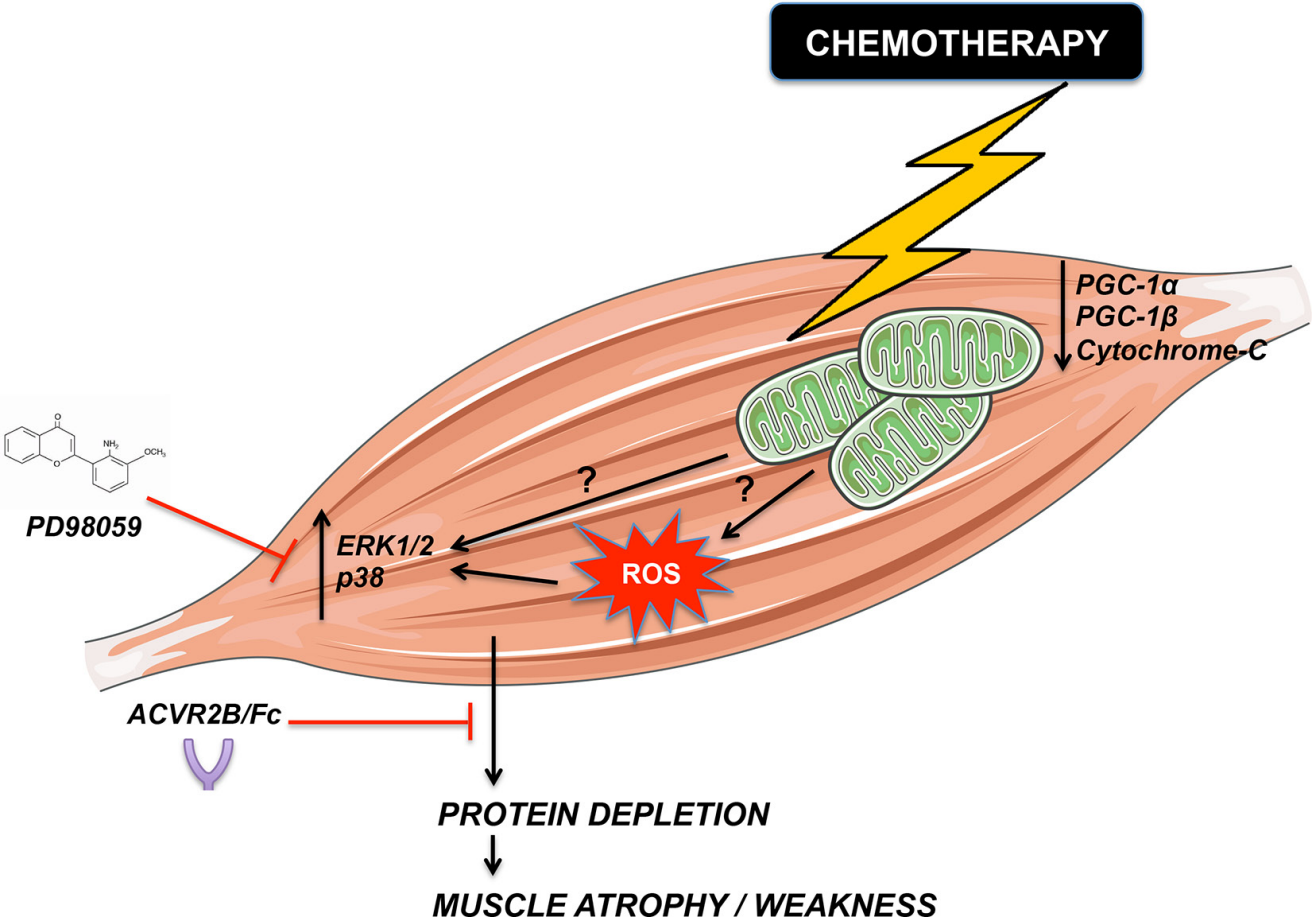
Representative model of chemotherapy-dependent cachexia Based on our observations, chemotherapy causes mitochondrial depletion and (directly or indirectly) activation of ERK1/2 and p38 MAPKs-dependent pathways. Altogether, these alterations might lead to cachexia, characterized by loss of muscle mass and increased muscle weakness. Promoting muscle growth by taking advantage of ACVR2B/Fc or blocking ERK1/2 activation by means of the MEK1 pharmacologic inhibitor PD98059 can prevent muscle wasting.

The findings reported in the present study suggest that administration of antineoplastic drugs normally used for the therapy of colorectal cancer to healthy mice is associated with significant loss of muscle mass and reduced muscle strength, characterized by marked depletion in muscle mitochondrial content, reactive oxygen species (ROS) release, oxidative to glycolytic muscle fiber shifts and change in the expression of known modulators of skeletal muscle wasting, such as the MAPKs ERK1/2 and p38. Importantly, we also show that administration of ACVR2B/Fc or MEK1 inhibitors prevents Folfiri-associated muscle atrophy in *in vitro* conditions, thus supporting the idea that promoting muscle anabolism or preventing MAPKs activation may potentially counteract chemotherapy-derived cachexia (Figure 9).

Our observations are consistent with previously reported works, correlating the use of drugs with anti-proliferative activities with muscle atrophy and muscle weakness [6, 7]. It was previously proposed that chemotherapy may drive muscle atrophy due to the narrow therapeutic window of most antineoplastic agents, thereby leading to low efficacy in case of under-dosing and, conversely, to severe side effects in case of over-dosing [1]. Additionally, most cytotoxic agents are associated with greater drug toxicity in patients that are muscle-depleted or have lower lean body mass, a compartment that contributes to metabolize most of the chemotherapeutics [1, 20, 21]. Notably, according to the current medical practice, individual chemotherapy dosage is based on the assessment of body surface area (BSA), although the validity of this practice is currently being questioned and few alternative strategies aimed at reducing drug-associated toxicity have been proposed [33, 34]. Accordingly, the amount of anticancer compounds tested in the present study was adapted from previously published reports, shown to safely inhibit tumor growth, and from our own preliminary dose curve experiments [35–37]. Moreover, aware of the limitations associated with directly comparing *in vivo* experimental protocols to chemotherapy regimens used in clinical practice, by taking advantage of the Meeh equation, used to assess BSA in mice, we made sure that the amount of drugs to be delivered to the experimental animals was not exceeding clinically relevant concentrations [38–42]. To further support the absence of severe toxicity in our model, we also showed that chemotherapy caused only transient reduction in food consumption in the animals administered chemotherapy, although the overall food intake in the animals receiving chemotherapy was similar in the mice treated with the vehicle alone. This observation is also consistent with the effects normally reported in oncologic patients undergoing chemotherapy treatment [43]. Interestingly, the skeletal muscle proteolytic systems, commonly implicated in conditions characterized by muscle atrophy [44], did not seem to be responsible for the muscle wasting caused by chemotherapy in this and our models, as also supported by the absence of modulation of proteasome activity and ubiquitin ligases expression. Similarly, the involvement of the autophagy-associated muscle degradation seemed to be minimal, unlike what reported in other models associated with muscle depletion due to cancer growth [25, 45], thus suggesting that other mechanisms may be responsible for muscle loss following chemotherapy administration in the absence of a tumor.

In this study, we found higher levels of MAPKs, such as ERK1/2 and p38, as well as decreased AKT-dependent anabolism, particularly in association with Folfiri administration. MAPKs activation has been previously associated with muscle wasting [26, 46], and altered AKT-dependent anabolism and enhanced myostatin expression have both been implicated in cachexia [12, 13, 24, 47]. Of note, no p38 activation was reported in the muscle of Folfox-treated animals, unlike what we showed for the animals administered Folfiri. This might be explained by the difference in composition of the two chemotherapy regimens (e.g. CPT-11 in Folfiri *vs.* Oxaliplatin in Folfox) or by the dosing schedule utilized (e.g. animals were treated once a week with Folfox *vs.* twice a week with Folfiri). Nevertheless, absence of p38 modulation was already reported in the muscle of C26-bearing animals, whereas high levels of p-ERK1/2 were shown to be involved in regulating muscle loss in this experimental model [26]. Interestingly, evidence of a feedback loop between myostatin and MAPKs activation has been previously demonstrated and is supported by the finding that ACVR2B/Fc prevented the activation of both MEK1 and ERK1/2-dependent pathway in our model. Indeed, ERK1/2-mediated higher myostatin levels were reported in stress conditions, while myostatin was also shown to promote ERK1/2 activation, thus playing a role in repressing myoblast differentiation [48, 49]. In a previous study, wild type mice and mice bearing the Lewis Lung carcinoma showed hyperphosphorylation of p38 MAPK as well as inhibition of AKT signaling and activation of the myostatin pathway in response to different chemotherapeutics [11, 50]. However, our findings did not show modulation in the expression of myostatin or any other TGFβ-family member following treatment with either Folfox or Folfiri, thus suggesting that, at least in the present experimental model, other mediators might drive chemotherapy-dependent muscle atrophy.

The present study also found evidence of chemotherapy-related effects on sarcoplasmic and mitochondrial integrity. Ours is not the first evidence supporting the idea that mitochondrial alterations may contribute to drive muscle wasting in association with antineoplastic drugs. For example, previous studies showed that doxorubicin affects skeletal muscle mass and leads to loss of muscle tissue and increased fatigue and was also reported to augment the release of ROS by disrupting the mitochondrial energy metabolism [51]. This, in turn, is also known to promote the activation of MAPKs, such as ERK1/2 and p38, in the skeletal muscle [28]. In particular, our observations suggest that chemotherapy administration to normal animals significantly affects skeletal muscle mitochondrial content and is associated with increased release of ROS and with an increase in the number of glycolytic fibers. Similar skeletal muscle alterations, consistent with muscle weakness associated with mitochondrial ultrastructural abnormalities and reduced muscle oxidative capacity, are also reported in a setting of cancer cachexia [52]. Thus, these findings further support the idea that the concomitant use of antineoplastic drugs, by impinging on similar pro-atrophic mechanisms, might contribute to exacerbate cancer-associated muscle atrophy. Recently, it was also reported that pathologic TGFβ release in association with bone lesions mediates the occurrence of muscle weakness by causing alterations of Ca^2+^-dependent muscle force production in a metastatic breast cancer model [32]. However, at the time point that we analyzed we did not observe any change in the expression of TGFβ or any of the family members that we considered, at least in our experimental model. Ultimately, based on our findings, the occurrence of muscle weakness following chemotherapy administration may well result from a combined effect associated with the reduction of mitochondrial content and the loss of sarcoplasmic/structural proteins, as also suggested by detecting thinner Z-lines and I-bands [51, 53].

These changes were also consistent with the observations reported by the RNA-Seq analysis, suggesting down-regulation of mitochondrial genes, and by the assessment of the levels of mitochondrial markers such as PGC-1α, PGC-1β and Cytochrome C [51, 53]. Interestingly, despite the depletion of muscle mitochondria, whose involvement in the β-oxidation process is known, our analysis also identified increased expression of genes associated with lipid metabolism, such as Fabp1, Apoa2, Apob, Apoh. Altogether, this could well represent a compensatory mechanism associated with the fact that the muscle fibers aim to restore energy homeostasis by importing more lipids that, however, due to decreased mitochondrial capacity, cannot be broken down to acetyl-CoA and accumulate in muscle. This hypothesis is further supported by evidence showing that intramyocellular lipid droplets are detected in the presence of cancer and their number is proportional to weight loss and loss of adipose mass in other body compartments [54]. In our study, the peroxisome proliferator-activated receptor γ co-activator 1 (PGC-1) family of transcriptional co-activators have been known as central regulators of energy homeostasis in skeletal muscle and other tissues for quite some time. PGC-1α is a central mediator of muscle homeostasis and seems to be affected by almost all the signaling pathways that are activated in the contracting muscle fibers [55]. On the other hand, little is known about the role played by PGC-1β in skeletal muscle function, although it was reported to contribute to ameliorating lipid-induced insulin resistance and to reduce oxidative stress in skeletal muscle [56]. Interestingly, these sarcoplasmic and mitochondrial alterations mirror similar derangements observed in the muscle of tumor-bearing animals. These mice showing profound morphological changes at the sarcoplasmic levels were also shown to account for alterations resulting in energy insufficiency and significant muscle weakness associated with cancer cachexia [53, 57], thus further supporting the idea that similarities between chemotherapy-associated cachexia and tumor-derived cachexia exist. Indeed, based on the gene expression profiling, several genes encoding for acute phase response proteins, such as albumin and fibrinogens, are up-regulated in the muscle of Folfiri-treated animals. Therefore, this evidence is consistent with our previous findings reported in the muscle of C26-bearing animals, suggesting that skeletal muscle protein breakdown may contribute to the activation of a systemic acute phase response and to the sustainment of an overall inflammatory state [58].

Although the importance of the relationship between muscle mass *vs.* chemotherapy response/tolerability has been investigated for quite some time, no treatments have been approved thus far to minimize or prevent cancer therapy toxicities [1, 20, 21, 59]. Several pieces of evidence are available to suggest that counteracting muscle wasting by enhancing muscle anabolism or blocking muscle catabolism may not only improve quality of life and prolong survival in the presence of a tumor, but may also contribute to reduce chemotherapy toxicity, thus allowing to tolerate higher and more effective doses of drug [11, 18, 60]. In the present experimental work, although we were not able to detect any change in myostatin levels in the muscle of chemotherapy-treated animals, we tested the administration of ACVR2B/Fc, a well-known myostatin inhibitor, as a powerful tool aimed at mitigating chemotherapy toxicity in C2C12 cultures by promoting muscle anabolism *per se*. Notably, pharmacologically improving muscle mass by administration of ACVR2B/Fc was also shown to rescue C26-associated cachexia and significantly prolong survival [12, 13, 18, 19]. Along the same line, the use of the anabolic agent ghrelin was shown to be partially successful in preventing cisplatin-associated muscle atrophy by reducing the inflammatory state and counteracting the activation of numerous pro-catabolic pathways [11, 61]. In a previous study, Fanzani et al. [62] showed that promoting muscle anabolism through insulin administration or delivery of a constitutively activated isoform of AKT counteracts chemotherapy-induced protein breakdown and restores myofiber size. Analogously, supplementation with fish oil and selenium was reported to be beneficially effective in preventing inflammation- and myostatin-associated muscle atrophy in tumor-bearing mice undergoing anticancer therapy, thus suggesting that nutrition supplementation might be taken into consideration in association with chemotherapy [60]. Similarly, our observations show that PD98059 selectively inhibits MEK1 and blocks activation of the downstream ERK1/2 pathways, thus also preventing muscle atrophy. Consistently, blockade of the ERK1/2-dependent signaling mediated by MEK1 pharmacologic inhibitors was previously shown to promote anabolism and to protect muscle mass in cancer cachexia, as well as in patients affected with cholangiocarcinoma [26, 63].

In conclusion, muscle wasting and muscle weakness are not only debilitating and pervasive complications of cancer development that generally contribute to an overall worsening of the quality of life of cancer patients, but may also be ascribed to chemotherapy-associated side effects, resulting in diminished physical activity, reduced tolerance to anticancer therapies and overall increased morbidity [9]. Our study design, not resembling completely the clinical scenario, where only subjects affected with cancer typically receive chemotherapy, does not take into consideration the complexity of the interactions between tumor- and chemotherapy-driven mediators, thus limiting our findings to those signaling pathways that are activated exclusively in the presence of anticancer drugs. Moreover, the administration of chemotherapy to 8-week old mice may seem to affect the normal tissue growth in animals that, although sexually mature, at this age have not reached full muscle development. Regardless, our observations prove that the chemotherapy treatment does not affect the overall body growth, as also supported by the analysis of the tibia length (comparable in both vehicle- and Folfiri-treated animals) and by the assessment of lean tissue mass (showing only a moderate, but not significant, growth in the vehicle-treated animals between day 0 and day 35). Nevertheless, our model system was able to isolate mediators of muscle wasting, as well as morphologic and functional alterations directly associated with the administration of antineoplastic drugs, therefore addressing a gap of knowledge that had not been investigated thus far. Importantly, our findings further support the idea that enhancing muscle mass and preventing chemotherapy-derived cachexia may represent a novel strategy aimed to improve patients' tolerance to anticancer drugs, thus also improving quality of life and increasing the chances for cure and tumor-free survival.

## MATERIALS AND METHODS

### Ethic statement

All animal experiments were conducted with the approval of the Institutional Animal Care and Use Committee at Indiana University School of Medicine and were in compliance with the National Institutes of Health Guidelines for Use and care of Laboratory Animals and with the ethical standards laid down in the 1964 Declaration of Helsinki and its later amendments.

### Animals

CD2F1 male mice (*n* = 8; Harlan, Indianapolis, IN) were administered intraperitoneally (i.p.) Folfiri (5-fluorouracil, leucovorin, CPT-11) or Folfox (5-fluorouracil, leucovorin, oxaliplatin) for up to 5 consecutive weeks. All drugs were purchased from Sigma Aldrich (St. Louis, MO). The dosing schedule is shown in Table S2. Control mice received an equal volume of vehicle. The animals were weighed daily and their food consumption was recorded. At time of sacrifice, several tissues were collected, weighed, snap frozen in liquid nitrogen and stored at −80°C for further studies. The tibialis anterior muscle was frozen in liquid nitrogen-cooled isopentane, mounted in OCT and stored for morphological analyses.

### Cell culture

C2C12 skeletal myoblasts (ATCC, Manassas, VA) were grown in high glucose DMEM supplemented with 10% FBS, 100 U/ml penicillin, 100 mg/ml streptomycin, 100 mg/ml sodium pyruvate, 2 mM L-glutamine, and maintained at 37°C in 5% CO_2_. Differentiation to myotubes was induced by shifting confluent cultures to DMEM supplemented with 2% horse serum and replacing the medium every other day for 5 days. At 5 days, myotubes were exposed to different Folfiri (50 μg/ml 5-FU, 10 μg/ml Leucovorin, 20 μg/ml CPT-11; Sigma Aldrich, St. Louis, MO), in combination with either PD98059 (20 μM; Selleckchem, Houston, TX) or ACVR2B/Fc (10 μg/ml) or for up to 48h. ACVR2B/Fc protein expression from the stable Chinese hamster ovary (CHO) cells was induced with 100 nM Cadmium in serum-free CHO media, and ACVR2B/Fc protein was purified from the conditioned medium using protein A Sepharose, as shown in [64]. CHO-ACVR2B/Fc cells were a kind gift of Dr. See-Jin Lee (Johns Hopkins University, Baltimore, MD).

### Grip strength

The assessment of four-limb strength in experimental mice was performed by means of a commercially available automatic grip strength meter (Columbus Instruments, Columbus, OH) following the procedures described in [65]. Briefly, the absolute grip strength (expressed in grams) and the normalized grip strength (obtained by dividing the force value by the body weight of every single mouse) were recorded. In order to reduce procedure related variability, the same operator analyzed an average from several repeated peak force measurements in the same animal in a blind manner. For this assay, 5 measurements were performed, and the top three measurements were used for the analysis. Moreover, to avoid bias of habituation, the animals were tested once a week during the experimental period.

### *Ex-vivo* muscle contractility

Whole muscle contractility of the extensor digitorum longus (EDL) muscles was determined as previously described [32, 65]. EDL were dissected from hind limbs; stainless steel hooks were tied to the tendons of the muscles using 4–0 silk sutures, and the muscles were mounted between a force transducer (Aurora Scientific, Aurora, ON, Canada) and an adjustable hook. The muscles were immersed in a stimulation chamber containing O_2_/CO_2_ (95/5%) bubbled Tyrode solution (121 mM NaCl, 5.0 mM KCl, 1.8 mM CaCl_2_, 0.5 mM MgCl_2_, 0.4 mM NaH_2_PO_4_, 24 mM NaHCO_3_, 0.1 mM EDTA, 5.5 mM glucose). The muscle was stimulated to contract using a supramaximal stimulus between two platinum electrodes. Data was collected via Dynamic Muscle Control/Data Acquisition (DMC) and Dynamic Muscle Control Data Analysis (DMA) programs (Aurora Scientific). At the start of each experiment the muscle length was adjusted to yield the maximum force. The force–frequency relationships were determined by triggering contraction using incremental stimulation frequencies (0.5ms pulses at 1–150 Hz for 350ms at supramaximal voltage). Between stimulations, the muscle was allowed to rest for 3 min. At the end of the force measurement, the length (L_0_) and weight of the muscle was measured to facilitate determination of the specific force. Specific force is the absolute force normalized to the muscle the cross-sectional area, calculated as shown in [66]. The investigators were blinded to treatment of subjects.

### Body composition assessment

Whole body composition (e.g. lean and fat tissue content) was measured every 7 days over the whole experimental periods in un-anesthetized but physically restrained mice using an Echo Medical systems' EchoMRI-100 (EchoMRI, Houston, USA). Data were expressed as variations over the baseline values.

### Activity monitoring

Activity was assessed using non-invasive monitoring of horizontal and vertical movements by placing mice in Accuscan activity monitors. Briefly, mice were removed from their home cages and placed within the acrylic chambers of VersaMax AccuScan activity monitors. These activity monitors consist of four horizontal sensors and two vertical sensors, each containing 16 infrared beams set 2.5 cm apart. Animal activity was detected and measured automatically by interruption of the infrared beams. Activity was recorded for 5 min and the movement was expressed as number of counts. Mice were then returned to their home cages. Based on our experience 5 min recording is sufficient to detect significant differences in horizontal or vertical movements.

### Real-time quantitative polymerase chain reaction (qRT-PCR)

RNA from quadriceps was isolated using the miRNeasy Mini kit (Qiagen, Valencia, CA, USA) and following the protocol provided by the manufacturer. RNA was quantified by using a Synergy H1 spectrophotometer (Biotek, Winooski, VT, USA). RNA integrity was checked by electrophoresis on 1.2% agarose gel containing 0.02 mol/L morpholinopropanesulfonic acid and 18% formaldehyde. Total RNA was reverse transcribed to cDNA by using the Verso cDNA kit (Thermo Fisher Scientific, Waltham, MA, USA). Transcript levels were measured by Real-Time PCR (Light Cycler 96, Roche, Indianapolis, IN, USA) taking advantage of the TaqMan gene expression assay system (Life Technologies, Carlsbad, CA). In particular, expression levels for Atrogin-1 (Mm00499523_m1), MuRF-1 (Mm01185221_ m1), Fbxo21 (SMART; Mm01208074_ m1), Fbxo30 (MUSA1; Mm01191299_ m1), Fbxo31 (Mm00505343_m1), TGF-β1 (Mm1178820_m1), TGF-β2 (Mm00436955_ m1), myostatin (Mm01254559_m1), Activin A (Mm00434339_ m1), MyoD (Mm00440387_m1), Myogenin (Mm00446194_m1) and Pax-7 (Mm01354484_m1) were detected. Gene expression was normalized to TBP (Mm01277042_m1) levels using the standard 2^−ΔCT^ methods.

### Western blotting

Total protein extracts were obtained by lysing cell layers or homogenizing 100 mg quadriceps muscle tissue in RIPA buffer (150 mM NaCl, 1.0% NP-40, 0.5% sodium deoxycholate, 0.1% SDS, and 50 mM Tris, pH 8.0) completed with protease (Roche, Indianapolis, IN) and phosphatase (Thermo Scientific, Rockford, IL) inhibitor cocktails. Cell debris were removed by centrifugation (15 min, 14000 g) and the supernatant collected and stored at −80°C. Protein concentration was determined using the BCA protein assay method (Thermo Scientific, Rockford, IL). Protein extracts (30 μg) were then electrophoresed in 4–15% gradient SDS Criterion TGX precast gels (Bio- Rad, Hercules, CA). Gels were transferred to nitrocellulose membranes (Bio- Rad, Hercules, CA). Membranes were blocked with SEA BLOCK blocking reagent (Thermo Scientific, Rockford, IL) at room temperature for 1 hour, followed by an overnight incubation with diluted antibody in SEA BLOCK buffer containing 0.2% Tween-20 at 4°C with gentle shaking. After washing with PBS containing 0.2% Tween-20 (PBST), the membrane was incubated at room temperature for 1 hour with either Anti-rabbit IgG (H+L) DyLight 800 or Anti-mouse IgG (H+L) DyLight 600 (Cell Signaling Technologies, Danvers, MA). Blots were then visualized with Odyssey Infrared Imaging System (LI-COR Biosciences, Lincoln, NE). Optical density measurements were taken using the Gel-Pro Analyzer software. Antibodies used were pSTAT3-Y705 (#9145), STAT3 (#8768), p-Akt-S473 (#4060), Akt (#9272), p-ERK1/2 (p-p44/42 MAPK, T202/Y204) (#4370), ERK1/2 (p44/42 MAPK) (#4695), pMEK1/2 (S217/221) (#9154), MEK1/2 (#9126), p-P38 MAPK (T180/Y182) (#4511), P38 MAPK (#9212), Cytochrome C (#11940) from Cell Signaling Technologies (Danvers, MA), PGC-1α (#ab3242), PGC**-**1β (#ab176328) from Abcam (Cambridge, MA), Beclin-1 (#B6186), LC3B (#L7543) from Sigma Aldrich (St. Louis, MO), p62 (#610832) from BD Biosciences (Franklin Lakes, NJ) and α-Tubulin (#12G10) from Developmental Studies Hybridoma Bank (Iowa City, IA).

### Proteasome activity

Chymotrypsin-like proteasome activity in quadriceps muscle from mice treated with either Folfox or Folfiri was determined as shown in [67]. Briefly, the tissue was homogenized in 20 mM Tris–HCl, pH 7.2, containing 0.1 mM EDTA, 1 mM 2-mercaptoethanol, 5 mM ATP, 20% glycerol, and 0.04% (v/v) Nonidet P-40. Muscle homogenates were then centrifuged at 13,000 × g for 15 minutes at 4°C. The supernatant was collected and protein concentration determined. Protein extracts were then incubated for up to 60 minutes at 37°C in the presence of 40 μM Suc-Leu-Leu-Val-Tyr-7-AMC specific fluorogenic substrate (Sigma Aldrich, St. Louis, MO), with or without MG-132 proteasome inhibitor (100 μM; EMD Millipore, Billerica, MA) in 50 mM Hepes, pH 8.0, 5 mM EGTA buffer. Fluorescence was detected with a spectrofluorometer (350 nm excitation, 440 nm emission; Synergy H1, BioTek, Vinooski, VT). Proteasome extracts from quadriceps muscle excised from mice bearing the C26 tumor, known to activate proteasome-dependent muscle catabolism [68], were utilized as positive control. The activity, obtained by evaluating the release of free AMC, is expressed as units of proteasome activity per ml (U/ml), where one unit of proteasome activity is defined as the amount of proteasome which generates 1.0 nmol of AMC per minute at 37°C.

### RNA-sequencing library construction and analysis

Standard methods were used for polyA mRNA-seq library construction, EZBead preparation and Next-Gen sequencing, based on Life Technologies SOLiD5000xl system. Briefly, one microgram total RNA per sample was applied for library preparation. PolyA mRNA was first captured using the standard protocol of Dynabeads® mRNA DIRECT™ Micro Kit (Life Technologies, #61021). Following the enrichment of polyA mRNA, the cDNA library was prepared and barcoded per sample using the standard protocol of SOLiD Total RNA-seq Kit (Life Technologies, #4445374). Each barcoded library was quantified by Bioanalyzer High Sensitivity DNA chip (Agilent, #5067-4626) and pooled in equal molarity. EZBead preparation, bead library amplification, and bead enrichment were then conducted using Life Technologies EZ Bead™ E80 System (Life Technologies, #4453095). Approximately four hundred forty million library-enriched beads per lane were deposited onto a SOLiD5500xl FlowChip (6 lanes/chip). Finally sequencing by ligation was carried out using standard single-read, 5′-3′strand-specific sequencing procedure (75b-read) on SOLiD5500xl Sequencer. The resulting 75 bp solid reads were mapped to *Mus musculus* mm9 reference genome using in-house mapping pipelines that utilizes bfast-0.7.0a [69]. In brief, using our RNA-seq pipeline, low quality reads and reads mapped to rRNA/tRNAs were first discarded. The remaining reads were mapped to reference genome mm9 and a splice-junction library, respectively; the genomic and splice-junction library mapping were merged at the end. The gene based expression levels were calculated using bamutils from NGSUtils based on the RefSeq gene annotation of mm9 [70]. Differential expression of genes across different treatments was determined with edgeR [71]. Statistically significant differentially expressed genes (False Discovery Rate < 5%) between control and cachectic muscles were imported into Ingenuity Pathway Analysis (Qiagen, Valencia, CA) to identify significant pathways, upstream regulators, and causal networks. The data discussed in this publication have been deposited in NCBI's Gene Expression Omnibus (GEO) [72]and are accessible through GEO Series accession number GSE80473.

### Ultrastructural analysis (Transmission Electron Microscopy - TEM)

EDL muscles were fixed with 3% Glutaraldehyde in 0.1 M sodium cacodylate (SC) buffer (pH 7.3) and, subsequently, rinsed three times in 0.1 M SC buffer. Muscle specimens were then post-fixed with 1% osmium tetroxide (OsO4) in 0.1 M SC buffer for 60 minutes. After post-fixation, samples were rinsed three times in 0.1 M SC buffer and dehydrated through a graded series of alcohol. The specimens were then placed in pure acetone for 2 changes at 10 min each, followed by a 50:50 mixture of acetone and epoxy resin (Embed 812, EMS) for overnight on a rotating platform and finally into 100% epoxy resin for approximately 4 hours, under vacuum, then placed in fresh resin and polymerized overnight in a 60°C oven. Tissue blocks were sectioned at 80 nm on a Leica UCT ultramicrotome (Leica Biosystems, Inc., Buffalo Grove, IL) and picked up on copper grids. Sections were stained with a saturated solution of uranyl acetate in 50% Ethanol for 5 min and lead-citrate stained for 10–20 seconds. Grids were viewed using a FEI Tecnia G 12 Bio-Twin (Hillsboro, OR) and imaged with an AMT (Danvers, MA) CCD camera. Mitochondrial content was determined by quantifying the number and the size (minimum diameter) of each mitochondria per field. A total of 20 fields per condition were analyzed by taking advantage of the Image J software [73].

### Morphological studies (fiber size, IF, SDH staining)

For histology and morphometry of muscle, tibialis anterior muscles were rapidly excised and mounted in OCT and frozen in liquid nitrogen-cooled isopentane for histology as shown in [57]. All samples were observed under an Axio Observer Z1 motorized microscope (Zeiss, Oberchoken, Germany) and calibrated images were recorded for morphometric examination. For skeletal muscle analyses, 8 μm-thick cryosections of tibialis anterior muscles taken at the mid-belly were processed for Hematoxylin & Eosin or SDH (succinate dehydrogenase) staining. In particular, for the SDH staining sections were incubated for 30 min at 37°C with 1 mg/mL NTB (nitrotetrazolium blue chloride) and 27 mg/mL Na- succinate in PBS. Afterwards the slides were washed three times in PBS, mounted with glycerol and photographed at different magnifications. Oxidative (dark blue) and glycolytic (light blue) fibers were quantified in tibialis anterior muscle sections stained for SDH and expressed as percentage of the total number of fibers per field. About 20 micrographs per condition were examined. For determination of the cross-sectional area (CSA), expressed as fiber area, muscle fibers (*n* = 300– 500 per sample) were measured by tracing the perimeter of each individual fiber using a Cintiq pen tablet input device (Wacom, Vancouver, WA, USA) and Image J 1.43 software [73]. For analysis of C2C12 myotube size, cells were fixed in ice-cold acetone-methanol and incubated with an anti-Myosin Heavy Chain antibody (1:1000, Millipore, Billerica, MA, USA) and an AlexaFluor 488- or AlexaFluor 594-labeled secondary antibody (Invitrogen, Grand Island, NY, USA). Analysis of myotube size was performed by measuring the average diameter of long, multi-nucleate fibers (*n* = 100–200 per condition) avoiding regions of clustered nuclei on a calibrated image using the Image J 1.43 software [73].

### Assessment of ROS levels

ROS were measured in cytosolic fractions using 2′, 7′-dichlorofluorescin diacetate (DCFH-DA) as a probe. DCFHDA is a stable, non-fluorescent molecule that readily crosses the cell membrane and is hydrolyzed by intracellular esterases to non-fluorescent 2′, 7′-dichlorofluorescein (DCFH), which is rapidly oxidized, in the presence of peroxides, to highly fluorescent 2′, 7′-dichlorofluorescein (DCF). The DCF is then measured fluorimetrically, as shown in [74]. Results are expressed as units of fluorescence (UF)/μg protein.

### Statistical analysis

All results are expressed as means ± SEM. Western blots show independent samples and are representative of at least two trials. Significance of the differences was evaluated by unpaired *T*-test or analysis of variance (ANOVA) followed by Tukey's post-test. Difference was considered significant when *p* < 0.05.

## SUPPLEMENTARY MATERIALS


