# Supplementary material for: Chemotherapy-related cachexia is associated with mitochondrial depletion and the activation of ERK1/2 and p38 MAPKs
Source: Oncotarget. 2016 Jun 2;7(28):43442–60. doi: 10.18632/oncotarget.9779 (PMC5190036; doi:10.18632/oncotarget.9779)
Supplement: Supplementary file 1 [file oncotarget-07-43442-s001.pdf]

Chemotherapy-related cachexia is associated with mitochondrial depletion and the activation of ERK1/2 and p38 MAPKs

Supplementary Materials

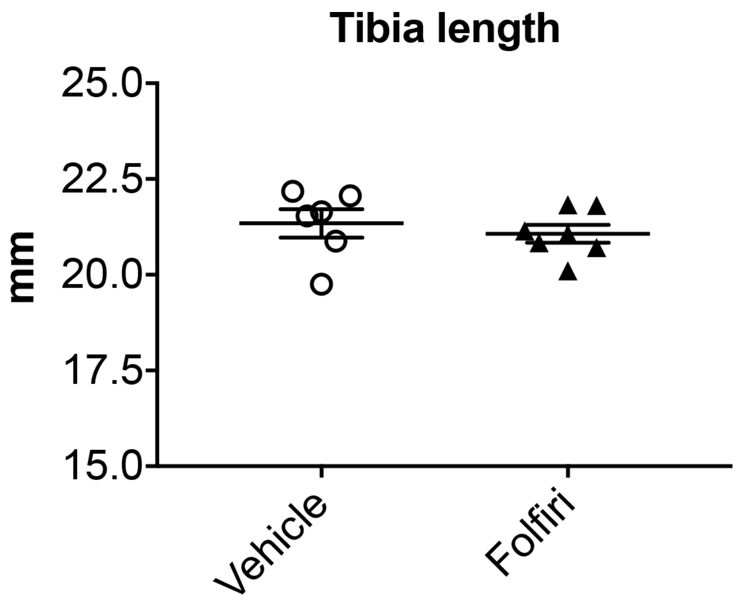

Supplementary Figure S1: Tibia length is unchanged in animals receiving chemotherapy. Tibia length in mice receiving either Folfiri or the control vehicle for up to 5 weeks was measured by taking advantage of a digital caliper. Data are reported on a scattered dot plot as Vehicle (n = 6) and Folfiri (n = 7) and expressed as means ± SEM.

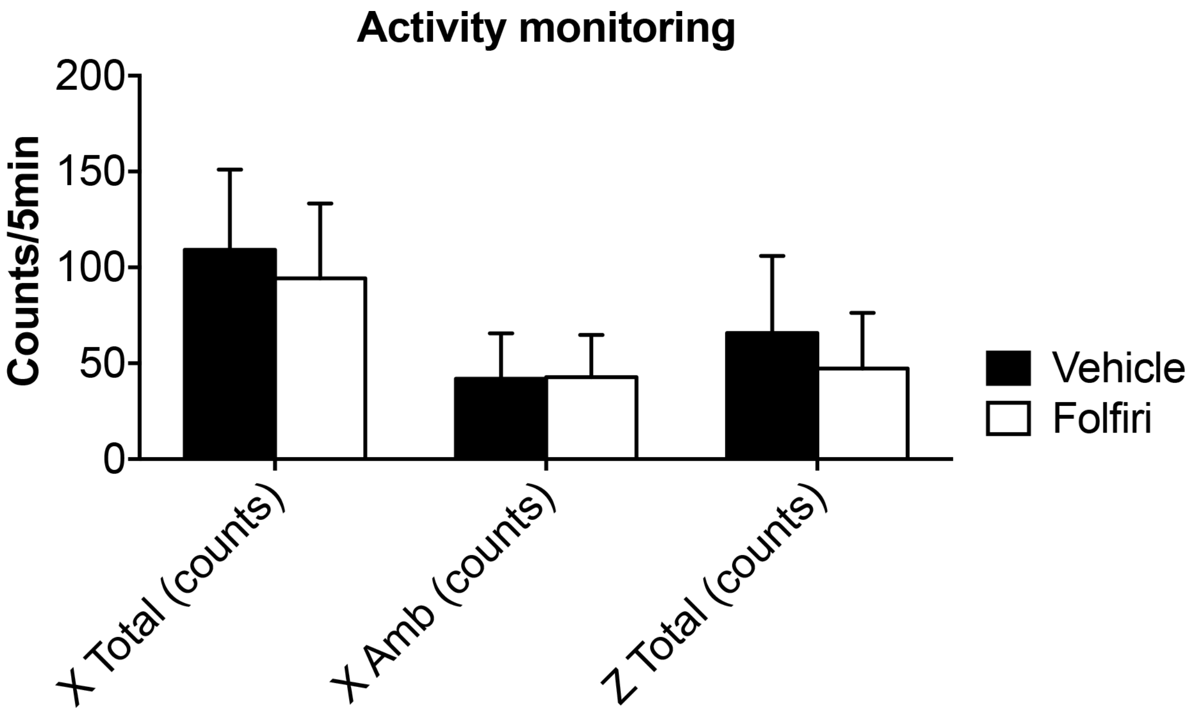

Supplementary Figure S2: Activity monitoring in mice exposed to Folfiri. Activity monitoring in animals administered chemotherapy was assessed by utilizing the VersaMax AccuScan system. Animal movements along the X and Z axes were detected. Data are reported as Counts/5 minutes and expressed as means ± SEM.

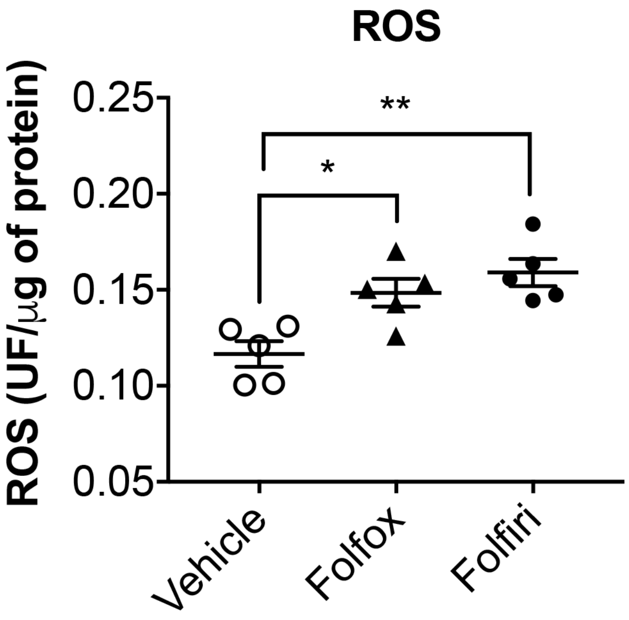

Supplementary Figure S3: Detection of ROS levels in the skeletal muscle of mice exposed to chemotherapy. Detection of ROS levels in the quadriceps muscle of mice exposed to Folfox or Folfiri ($n = 5$) for up to 5 weeks. Data are reported on a scattered dot plot and expressed as means ± SEM. Significance of the differences: *$p < 0.05$; **$p < 0.01$.

Supplementary Table S1: Next-Generation RNA-sequencing analyses

Gene	Log Fold Change	*p* value	FDR	Function
Ucp1	–6.4	2.66E-05	1.81E-02	Mitochondrial metabolism/biogenesis
Cidea	–4.6	1.36E-04	5.00E-02	Mitochondrial metabolism/biogenesis
Nnat	–3.3	7.05E-05	3.40E-02	Other
Hspa1b	–3.1	6.63E-05	3.40E-02	Heat shock response
Gyk	–2.8	5.51E-05	3.05E-02	Other
Fam107a	–2.3	1.29E-06	5.00E-03	Other
Chac1	–1.8	7.95E-06	1.28E-02	Notch signaling regulator
Acot2	–1.7	3.38E-05	2.18E-02	Mitochondrial metabolism/biogenesis
Fhl3	–1.2	7.12E-05	3.40E-02	Muscle cell proliferation/pluripotency
Prkcz	1.4	9.13E-05	3.92E-02	Other
Lrp2bp	1.5	1.20E-05	1.41E-02	Other
Sytl2	1.5	2.37E-05	1.70E-02	Other
Masp2	1.6	1.24E-04	5.00E-02	Other
Car9	1.6	1.02E-04	4.23E-02	Other
Scd2	1.6	5.24E-06	1.13E-02	Lipid metabolism/transport
Spon2	1.7	5.68E-05	3.05E-02	Member of the ERK1/2 signaling
Pax3	2.0	1.34E-04	5.15E-02	Muscle cell proliferation/pluripotency
Fam196b	2.2	3.96E-05	2.43E-02	Other
Hamp2	2.3	4.98E-05	2.92E-02	Other
Exoc3l4	2.8	1.26E-07	9.52E-04	Other
Apoh	3.0	1.37E-05	1.47E-02	Lipid metabolism/transport
Slc4a1	3.6	1.55E-06	5.00E-03	Ion transport
Ccl8	3.7	1.00E-05	1.31E-02	Chemokine/Inflammation
Apoa1	4.5	1.01E-05	1.31E-02	Lipid metabolism/transport
Mup6	4.6	4.99E-06	1.13E-02	Other
Alb	4.7	2.14E-05	1.70E-02	Acute phase response
Fga	4.9	7.28E-06	1.28E-02	Acute phase response
Ttr	5.1	2.35E-05	1.70E-02	Other
Pzp	5.1	7.67E-05	3.43E-02	Other
Fgb	5.2	7.72E-05	3.43E-02	Acute phase response
Apoa2	5.4	2.25E-05	1.70E-02	Lipid metabolism/transport
Apob	5.7	1.88E-05	1.70E-02	Lipid metabolism/transport
Fabp1	6.0	1.83E-05	1.70E-02	Lipid metabolism/transport
Dnah5	8.1	1.48E-07	9.52E-04	Energy metabolism/ATPase

Data expressed as Log Fold Change vs. Vehicle. Only genes associated with FDR ≤ 0.05 were included in the table.

Supplementary Table S2: Chemotherapy dosing schedule

<table>
<tr><th>Drug</th><th>mg/Kg</th><th colspan="2">Chemotherapy regimen & Dosing schedule</th></tr>
<tr><td>Oxaliplatin</td><td>6</td><td rowspan="3">FOLFOX
(Oxaliplatin administered 2h before 5-FU and Leucovorin)
i.p. once/week</td><td></td></tr>
<tr><td>5-FU</td><td>30</td><td rowspan="3">FOLFIRI
(CPT-11 administered 2h after 5-FU and Leucovorin)
i.p. twice/week</td></tr>
<tr><td>Leucovorin</td><td>90</td></tr>
<tr><td>CPT-11</td><td>24</td><td></td></tr>
</table>
